# Biochar and straw amendments drive microbial regulation of phosphorus dynamics in saline-irrigated cotton fields

**DOI:** 10.3389/frmbi.2025.1508717

**Published:** 2025-02-06

**Authors:** Yang Ye, Xiaowen Guo, Yueyao Li, Wei Min, Huijuan Guo

**Affiliations:** Department of Resources and Environmental Science, Shihezi University, Shihezi, Xinjiang, China

**Keywords:** saline water irrigation, biochar, straw, P transformation, functional genes

## Abstract

Saline water drip irrigation is a potential solution for addressing freshwater scarcity in arid regions. However, prolonged use can accumulate soil salinity and reduce phosphorus (P) availability. Biochar and straw amendments have been shown to alleviate these effects, but their mechanisms in regulating microbial genes involved in P transformation under long-term saline irrigation remain unclear. This study aimed to evaluate the impact of biochar and straw incorporation on soil microbial community structure and P availability in saline-irrigated cotton fields. Based on a 14-year field trial, three treatments were developed: saline water irrigation alone (CK), saline water irrigation with biochar (BC), and saline water irrigation with straw (ST). Results indicated that both amendments significantly enhanced soil water content, organic carbon, total P, available P, and inorganic P fractions (Ca_10_-P, Al-P, Fe-P, and O-P) while reducing soil electrical conductivity and Ca_2_-P and Ca_8_-P fractions. Biochar increased the relative abundance of Chloroflexi, Gemmatimonadetes, and Verrucomicrobia, while straw promoted Proteobacteria and Planctomycetota. Both treatments decreased the abundance of several P mineralization genes (e.g., *phoD*, *phoA*) and increased genes associated with P solubilization (e.g., *gcd*). Microbial populations and P cycling genes were shown to be tightly associated with soil characteristics, with Ca_2_-P and Al-P serving as important mediators, according to correlation studies. Generally, under long-term salty irrigation, biochar, and straw amendments reduced soil salinity, raised soil P availability, decreased the expression of phosphorus cycling-related microbial genes, and improved soil characteristics. These results made them excellent techniques for sustainable soil management.

## Highlights

- Long-term saline water irrigation reduces soil phosphorus (P) availability and increases soil salinity.
- Biochar and straw amendments improved soil P availability and reduced salinity accumulation.
- Biochar and straw significantly altered the structure and function of soil microbial communities.
- Key P-cycling genes (e.g., gcd, phnP and phnA) were upregulated, enhancing P solubilization and mineralization.
- Correlation analysis identified Ca2-P and Al-P as primary drivers of microbial and functional gene changes.

## 1 Introduction

The lack of freshwater in desert areas poses serious obstacles to agricultural output (Awaad et al., 2020). In Xinjiang, a typical desert region, annual evaporation is more than precipitation, resulting in a serious lack of freshwater resources for irrigation of agriculture. However, Xinjiang has abundant saline water resources, making developing and utilizing saline water for agricultural purposes a viable solution to address freshwater shortages (Cheng et al., 2021). Cotton, one of China’s most important cash crops, plays a vital role in agricultural development. Being a salt-tolerant crop, its yield and quality are influenced by soil salinity and nutrient availability (Atta et al., 2023; Vermue et al., 2013). Saline water drip irrigation has been widely adopted as an efficient water-saving method in many regions (Hu et al., 2023). However, long-term saline water drip irrigation can lead to salt accumulation, negatively affecting soil physicochemical properties, disrupting soil microbial communities (Zhang W et al., 2019), reducing soil fertility (Guo et al., 2023; Zhu et al., 2021), and impairing soil phosphorus (P) dynamics, which subsequently influences cotton growth (Guo et al., 2019).

Soil P is a critical nutrient for plant growth, and its bioavailability directly affects crop yield and quality (Pang et al., 2024). The transformation and availability of soil P are primarily regulated by microorganisms through complex biological processes, including organic P mineralization, inorganic P solubilization, phosphate fixation, and biological P uptake (Chen et al., 2023; Andriamananjara et al., 2016). In the process of organic phosphorus mineralization, microorganisms secrete acid and alkaline phosphatases that hydrolyze organic phosphorus compounds, converting them into inorganic phosphorus (e.g., phosphate) that can be absorbed by plants (Wan et al., 2022). The expression of alkaline phosphatase is regulated by homologous genes, including *phoA*, *phoD*, and *phoX*, which are encoded by bacteria (Tan et al., 2013). These genes play significant roles in both terrestrial and aquatic ecosystems (He et al., 2020). Among them, the *phoD* gene has been extensively studied in terrestrial ecosystems due to its strong correlation with soil organic phosphorus mineralization capacity and available phosphorus levels. It is widely used as a molecular marker for assessing the diversity and functionality of soil microbial communities (Hu et al., 2018). Generally, the abundance of the *phoD* gene is negatively correlated with available phosphorus in soils, as phosphorus deficiency often stimulates microbial communities to secrete more phosphatases (Fraser et al., 2015). However, some studies have suggested that the abundance of the *phoD* gene may exhibit positive or no correlation with available phosphorus (Hu et al., 2018). Additionally, some microorganisms release organic acids, such as citric acid and oxalic acid, to dissolve phosphate minerals in the soil, thereby increasing the availability of phosphorus (Aguiar et al., 2024). The *gcd* gene, which encodes glucose dehydrogenase, plays a particularly important role in this process as it facilitates the production of gluconic acid, a strong organic acid capable of effectively dissolving inorganic phosphate minerals, thereby making them more accessible for plant uptake (Zeng et al., 2016). However, due to high salt stress and low moisture conditions in arid regions, the availability of soil P is often limited, posing challenges in meeting the nutrient demands of crops (Ahmad et al., 2021). High salinity levels can suppress soil microbial activity; however, soil microbes play a key role in the P cycle and are crucial for regulating soil P availability and maintaining plant productivity (Lychuk et al., 2019). Due to natural selection under continuous salt stress, microbes in high-salinity environments often develop greater salt tolerance (Rath and Rousk, 2015). Studies have shown that there is a beneficial relationship between soil P efficacy and salinity. At moderate salinity levels, the abundance of P-cycling microbial communities can be increased, promoting microbial P dissolution and mineralization (Hu et al., 2023). Therefore, exploring effective strategies to regulate soil P transformation is of significant importance for the sustainable development of agriculture under saline water irrigation in arid regions.

Biochar and straw, as soil amendments, have demonstrated significant potential in improving soil structure, enhancing microbial activity, and increasing the bioavailability of P (He et al., 2020; Pu et al., 2023). The high specific surface area and porous structure of biochar enable it to adsorb harmful substances, alleviate soil salt stress, and promote P dissolution and transformation by releasing organic acids and altering soil pH (Pastore et al., 2020). Returning straw to the soil increases soil organic matter content, providing abundant carbon sources and habitats for microorganisms, while also enhancing soil enzyme activity, further promoting the P cycling process (Ji et al., 2024; Lai et al., 2023). Studies have shown that biochar and straw incorporation not only reduce the inhibitory effects of salinity on soil microbial activity but also optimize the biological transformation of soil P by regulating the expression of functional genes related to P transformation, such as P genes and organic P mineralization genes (Yang and Lu, 2022). However, current research on the effects of biochar and straw amendments on the microbial mechanisms of soil P transformation under saline water irrigation conditions in arid regions is still limited.

This study is based on a long-term saline water irrigation cotton field experiment, systematically evaluating the effects of biochar and straw return on soil physicochemical properties, inorganic P component distribution, and P cycling microbial communities. We hypothesize that: (1) Long-term saline water irrigation increases soil salinity and reduces the availability of soil P. However, biochar and straw incorporation mitigate surface soil salt accumulation caused by long-term saline water drip irrigation, enhances soil phosphorus availability, and increases the potential for available phosphorus. (2) Biochar and straw return alter the composition of P-cycling microbial communities, driving the expression of P-related functional genes to adapt to the changed environment. The application of biochar and straw significantly reduced the abundance and diversity of microbial communities involved in phosphorus cycling, as well as the abundance of functional genes. The main objectives of this study were to evaluate the impact of biochar and straw amendments on soil physicochemical properties and the distribution of inorganic P fractions. Additionally, the study aimed to investigate changes in the abundance, diversity, and composition of P-cycling microbial communities, along with their associated functional gene responses to biochar and straw incorporation. Furthermore, the study sought to establish links between the functional genes of P-cycling microorganisms, soil physicochemical properties, and inorganic P fractions, thereby providing a comprehensive understanding of microbial-mediated P-cycling processes. Ultimately, this research aims to offer a theoretical foundation for optimizing soil P management practices under long-term saline water drip irrigation conditions in arid regions.

## 2 Materials and methods

### 2.1 Experimental site and design

The experiment was conducted in 2023 at the Agricultural College of Xinjiang Shihezi University (44°18′N, 86°02′E), which has a temperate continental climate with annual precipitation of 180-270 mm and evapotranspiration of 1,000-1,600 mm. The soil was loamy irrigated gray desert soil, and the crop was cotton (‘Xinlu Early 74’). In 2009, the initial soil properties were measured as follows: bulk density of 1.3 g·cm^−3^, pH of 7.9, electrical conductivity of 0.13 dS·m^−1^, organic matter content of 16.8 g·kg^−1^, total nitrogen content of 1.1 g·kg^−1^, available phosphorus content of 25.9 mg·kg^−1^, and available potassium content of 253 mg·kg^−1^. The study continued a 14-year saline water irrigation experiment (8.04 dS·m^−1^, NaCl: CaCl_2_ = 1:1). Since 2019, the annual application rates of biochar (BC) and straw (ST) have been set at 3.7 t·hm^−2^ and 6 t·hm^−2^, respectively. These rates were determined based on the full return of cotton straw to the field. Specifically, the shredded cotton straw biomass amounts to 6 t·hm^−2^, which, when converted into biochar through pyrolysis, corresponds to an equivalent application rate of 3.7 t·hm^−2^. Treatments included: (1) saline water drip irrigation (CK), (2) CK + biochar (BC), and (3) CK + straw (ST) in a randomized block design with three replicates (total: nine plots, 25 m² each). Cotton was sown on May 1st with a density of 222,000 plants·hm^−2^ under mulching. Irrigation (total 450 mm) was applied over nine sessions from June to August. Fertilizers included urea (360 kg·hm^−2^, split in six applications), P_2_O_5_ (105 kg·hm^−2^), and K_2_O (60 kg·hm^−2^) applied as basal. Other management followed local practices.

### 2.2 Soil sample collection and processing

Soil samples were collected from the 0-20 cm plow layer during the cotton bolling stage in 2023. After clearing surface debris with a clean shovel, three samples were taken from each treatment plot, combined, and passed through a 2 mm sieve to remove plant roots, leaves, crop residues, stones, and any non-soil particles. A portion of the fresh soil was immediately placed in an icebox, transported to the laboratory, and stored at -80°C for soil microbial community metagenomic sequencing. The remaining soil was air-dried, ground, sieved through a 1 mm mesh, and stored to analyze soil physicochemical properties and soil inorganic P fractions.

### 2.3 Soil sample analyses

Soil physicochemical properties and inorganic phosphorus fractions were analyzed to assess soil quality. Soil bulk density was determined using the core method. Soil water content (SWC) was determined using the gravimetric method by oven drying. Soil electrical conductivity (EC) and pH were measured using a conductivity meter (MP522, Shanghai Precision Scientific Instrument Co., China) and a pH meter, respectively, with water-to-soil ratios of 5:1 for EC and 2.5:1 for pH. Total organic carbon (TOC) was quantified using a TOC analyzer (Multi N/C 2100, Analytikjena, Germany), while total phosphorus (TP) was assessed using the HClO_4_-H_2_SO_4_ digestion method. Total nitrogen (TN) was determined using the Kjeldahl method. Available phosphorus (AP) was extracted using NaHCO_3_ and analyzed via the molybdenum-antimony colorimetric method. Available potassium (AK) was measured by ammonium chloride extraction followed by flame photometry. The inorganic phosphorus (P) fractions in soil were determined using the sequential extraction method described by Jiang and Gu (1989). A 1.000 g air-dried soil sample was placed in a 50 ml centrifuge tube, and sequentially extracted with six different extracting solutions. The P concentration in each extract was measured using the molybdenum-antimony colorimetric method at 700 nm.

### 2.4 DNA extraction and metagenomic sequencing

DNA was extracted from soil samples stored at -80°C using the Illumina NovaSeq Reagent Kits, following the manufacturer’s protocol. The extracted genomic DNA was verified through 1% agarose gel electrophoresis, and high-quality DNA fragments were stored for further processing. The DNA was sheared into 400 bp fragments using a Covaris M220 ultrasonicator, and the PE libraries were constructed using the NEXTFLEX Rapid DNA-Seq Kit (USA). Sequencing was performed on the Illumina HiSeq X-ten platform (Illumina, USA) using the PE150 strategy by Meiji Biotech (Shanghai, China). The raw sequencing data has been submitted to the NCBI database (BioProject ID: PRJNA1200914). The quality of the raw reads was checked using Fastp (v0.20.0, https://github.com/OpenGene/fastp) to filter out low-quality sequences, retaining only high-quality reads for downstream analysis. High-quality reads were assembled using MEGAHIT (v1.1.2, https://github.com/voutcn/megahit), a software based on the De-Bruijn graph principle, to generate contigs by aligning overlapping sequences. Contigs longer than 350 bp were selected for further analysis. Open Reading Frames (ORFs) were predicted using Prodigal (v2.6.3, https://github.com/hyattpd/Prodigal) software, and sequences were translated into amino acid sequences. Redundancy was minimized using CD-HIT (v4.6.1, http://www.bioinformatics.org/cd-hit/) software, clustering sequences at 95% identity and 90% coverage, with the longest sequence in each cluster chosen as the representative. High-quality reads from each sample were aligned to the non-redundant gene set using SOAPaligner (soap2.21release, https://github.com/ShujiaHuang/SOAPaligner) software, and the gene abundance in each sample was calculated accordingly. Final contigs (Scaftigs) were annotated and functionally categorized using the KEGG (Kyoto Encyclopedia of Genes and Genomes, http://www.genome.jp/kegg) database, enabling comprehensive gene prediction and species identification.

### 2.5 Data analysis

Data were organized using Microsoft Excel 2016 and analyzed with IBM SPSS 21.0 using Duncan’s test (*P*<0.05). Heat maps for P-cycling genes were created with MetaboAnalyst 6.0, and Alpha and Beta diversity were calculated using the vegan package in R (v4.1.3). Correlation analyses were conducted in Origin 2021 to identify key environmental factors influencing species and gene expression. Results are presented as mean ± standard deviation.

## 3 Results

### 3.1 Response of soil physicochemical properties to biochar and straw amendments

The incorporation of biochar (BC) and straw (ST) into the soil significantly enhanced soil water content (SWC), soil organic carbon (SOC), total phosphorus (TP), and available phosphorus (AP) compared to the control (CK) under long-term saline water drip irrigation conditions (**Table 1**). Additionally, both amendments resulted in a notable reduction in soil electrical conductivity (EC1:5), indicating decreased soil salinity. Although changes in soil pH were relatively minor, a slight increase was observed following the addition of BC and ST. These findings demonstrate that biochar and straw effectively improve soil quality and reduce salinity stress in saline irrigation systems.

**Table 1:** Soil physicochemical properties under different amendment treatments.

Treatment	SWC (%)	EC_1:5_ (dS·m^-1^)	pH	SOC (g·kg^-1^)	TP (g·kg^-1^)	AP (mg·kg^-1^)
CK	16.47 b	1.43 a	7.65 a	19.86 c	1.16 c	25.62 c
BC	17.75 a	1.35 b	7.70 a	23.21 b	1.22 b	32.16 b
ST	18.08 a	1.32 b	7.67 a	26.21 a	1.26 a	36.16 a

Different lowercase letters indicate significant differences between the means of values in the same column (*P* < 0.05). CK, long-term saline water drip irrigation; BC, biochar return under long-term saline drip irrigation conditions; ST, straw return under long-term saline drip irrigation conditions.

### 3.2 Soil inorganic P fractions

**Figure 1:**
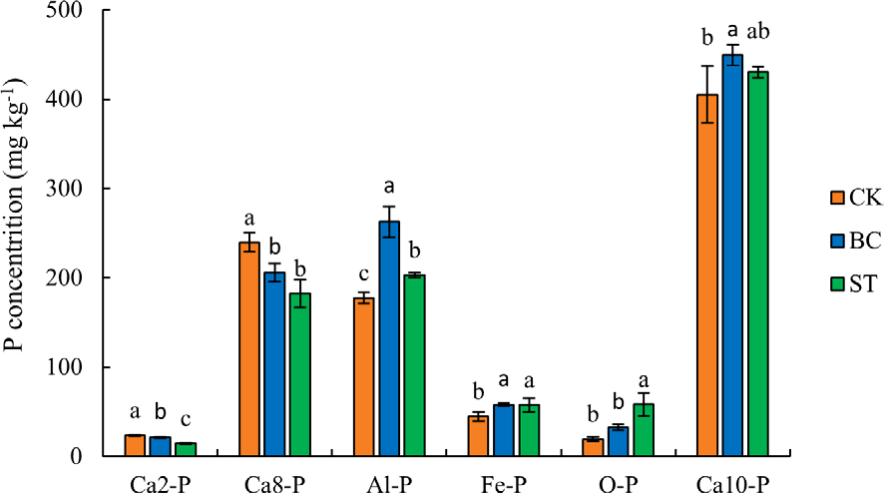
Soil inorganic P fractions under biochar and straw returned conditions. Columns represent mean values ± standard error (n = 3). Different lowercase letters above the columns indicate significant differences between treatments within the same P fraction (*P* < 0.05). CK, control under long-term saline water drip irrigation; BC, biochar amendment under saline irrigation; ST, straw amendment under saline irrigation.

The results presented in **Figure 1** show that biochar (BC) application significantly increased the soil content of aluminum-bound phosphorus (Al-P), iron-bound phosphorus (Fe-P), and hydroxyapatite (Ca_10_-P) by 47.9%, 29.5%, and 10.9%, respectively, compared to the control (CK). However, BC significantly reduced the content of more soluble calcium-bound phosphorus fractions, such as dicalcium phosphate (Ca_2_-P) and octacalcium phosphate (Ca_8_-P), by 9.2% and 14.1%, respectively. Similarly, straw (ST) application significantly elevated the concentrations of Al-P, Fe-P, and occluded phosphorus (O-P) by 14.3%, 28.5%, and 198.7%, respectively, but markedly decreased Ca_2_-P and Ca_8_-P content by 37.8% and 24.0%, respectively. Among the various soil inorganic phosphorus (P) fractions, hydroxyapatite (Ca_10_-P) was the predominant form, accounting for approximately 45% of the total inorganic P, followed by Al-P, Ca_8_-P, Fe-P, O-P, and Ca_2_-P.

### 3.3 Soil P transformation microbial community diversity

**Figure 2:**
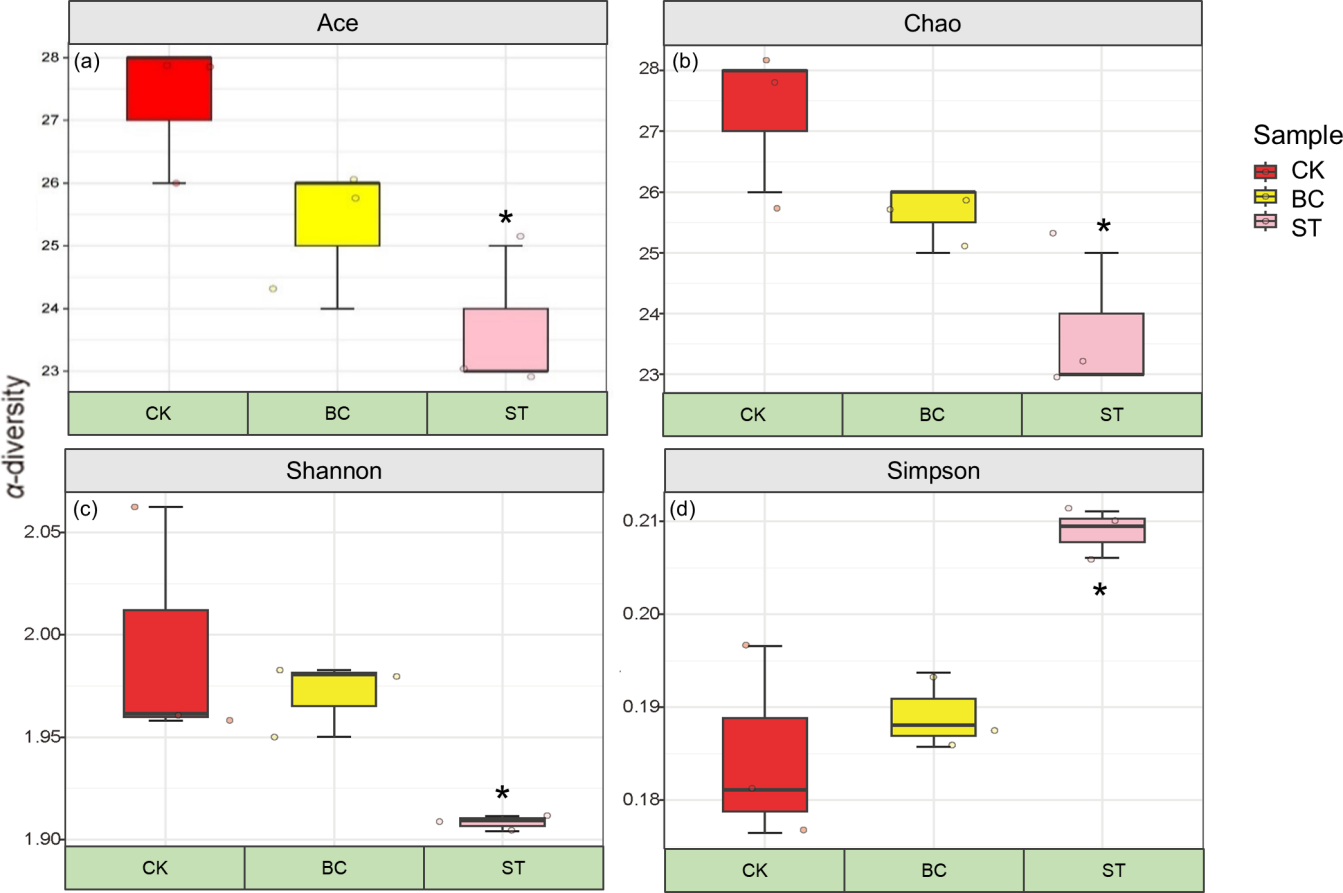
*α*-diversity indices of soil phosphorus (P) transformation microorganisms under different amendment treatments. Asterisks (*) indicate significant differences at *P* < 0.05. CK, control under long-term saline water drip irrigation; BC, biochar amendment under saline irrigation; ST, straw amendment under saline irrigation. In the figures, **(A–D)** represent the Ace, Chao, Shannon, and Simpson indices, respectively.

**Figure 2** shows that both biochar (BC) and straw (ST) amendments resulted in a decreasing trend in the Ace, Chao, and Shannon indices, while the Simpson index exhibited an increasing trend compared to the control (CK) treatment. The changes observed in all ST treatments were statistically significant. These results suggest that both BC and ST reduced the richness and diversity of the soil microbial community involved in phosphorus (P) transformation processes under saline water drip irrigation conditions.

**Figure 3:**
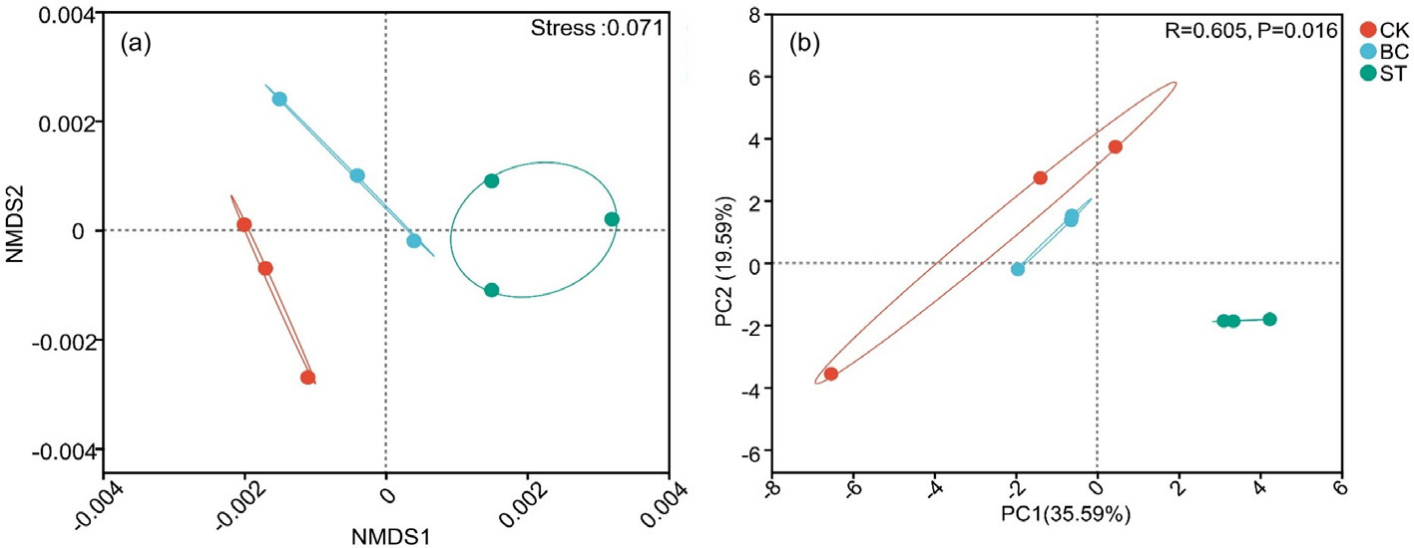
NMDS and PCA analyses of soil phosphorus (P) transformation microbial communities under different amendment treatments. The ellipses represent 95% confidence intervals around each treatment group. CK, control under long-term saline water drip irrigation; BC, biochar amendment under saline irrigation; ST, straw amendment under saline irrigation. In the figures, **(A, B)** represent the NMDS and PCA analyses, respectively.

The soil phosphorus (P) cycling microbial community structure was examined using Non-Metric Multidimensional Scaling (NMDS) (**Figure 3A**) and Principal Component Analysis (PCA) (**Figure 3B**). The NMDS analysis showed a stress value of less than 0.05, indicating a reliable representation of community differences. With a cumulative contribution of 55.18%, the first two principal components in the PCA analysis, PCA1 and PCA2, accounted for 35.59% and 19.59% of the total variation, respectively. The microbial communities under saline water drip irrigation in cotton fields were shown to be considerably affected by both biochar and straw amendments, as evidenced by the unique microbial communities seen in the CK treatment compared to the BC and ST treatments.

### 3.4 Soil P microbial community dynamics

**Figure 4:**
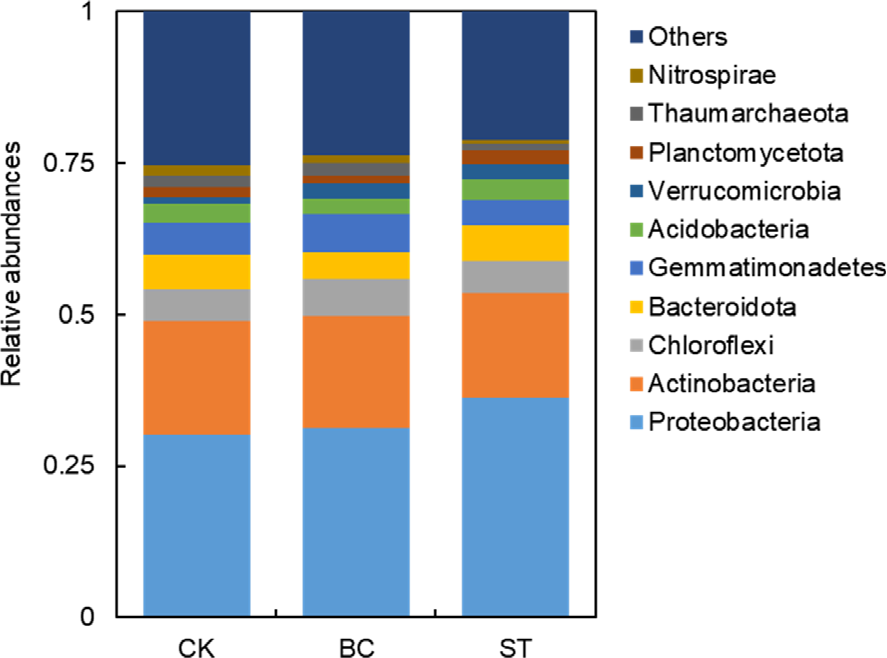
Illustrates the relative abundance of the top 10 microbial phyla involved in soil phosphorus (P) transformation under biochar (BC) and straw return (ST) treatments compared to the control (CK). The abbreviations are CK, control with long-term saline drip irrigation; BC, biochar under saline drip irrigation; ST, straw under saline drip irrigation.

The community composition of soil phosphorus (P) transformation microorganisms at the phylum level is illustrated in **Figure 4**. The predominant phyla included Proteobacteria (30.64%–36.55%), Actinobacteria (17.18%–19.09%), Chloroflexi (5.21%–6.13%), Bacteroidota (4.43%–5.86%), and Gemmatimonadetes (4.19%–6.32%), each with an average relative abundance exceeding 5%. Collectively, these phyla accounted for 67.59% (ranging from 66.25% to 69.24%) of the total microbial sequences. Biochar (BC) treatment significantly reduced the sequence abundance of Actinobacteria, Bacteroidota, Acidobacteria, and Planctomycetota by 1.5%, 24.0%, 18.7%, and 28.4%, respectively. Conversely, BC application markedly increased the relative abundance of Proteobacteria (3.1%), Chloroflexi (17.7%), Gemmatimonadetes (15.3%), Thaumarchaeota (13.1%), and Verrucomicrobia (163.9%). In contrast, straw treatment (ST) significantly lowered the relative abundance of Actinobacteria, Gemmatimonadetes, and Thaumarchaeota by 10.0%, 23.5%, and 51.0%, respectively, compared to the control (CK). However, ST notably increased the abundance of Proteobacteria (19.3%), Chloroflexi (4.8%), Acidobacteria (4.0%), Planctomycetota (52.5%), and Verrucomicrobia (157.7%).

### 3.5 Functional metabolism of soil P-transformation microorganisms

**Figure 5:**
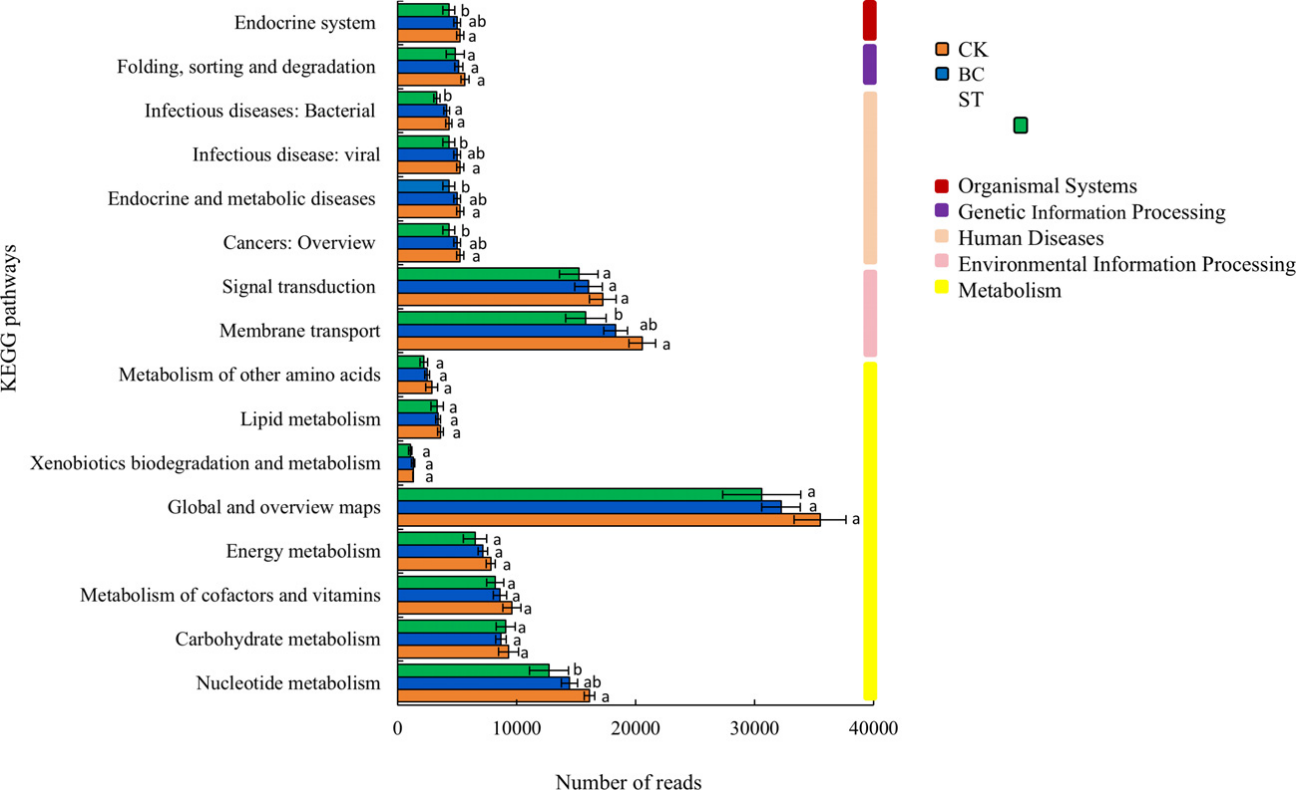
Functional annotation of KEGG for soil P transformation under biochar and straw returned conditions. Different lowercase letters indicate significant differences between the means of values in the same column (*P* < 0.05). Columns with bars represent the mean ± standard error (n= 3). CK, long-term saline water drip irrigation; BC, biochar return under long-term saline drip irrigation conditions; ST, straw return under long-term saline drip irrigation conditions.

The KEGG functional annotation analysis of soil phosphorus (P) transformation microorganisms (**Figure 5**) identified five primary categories and sixteen secondary functional groups, with metabolic functions being the most prevalent, constituting 50% of the total. Among the metabolic pathways, the highest abundance was observed in the “Global and overview maps” category, followed by pathways involved in “Nucleotide metabolism,” “Carbohydrate metabolism,” “Metabolism of cofactors and vitamins,” “Energy metabolism,” “Lipid metabolism,” “Metabolism of other amino acids,” and “Xenobiotics biodegradation and metabolism.” Besides metabolic pathways, microbial functions related to P cycling were also enriched in “Signal transduction” and “Membrane transport” pathways. Overall, the abundance of all P-related functional groups in soils treated with biochar and straw showed a declining trend under saline water drip irrigation conditions.

**Figure 6:**
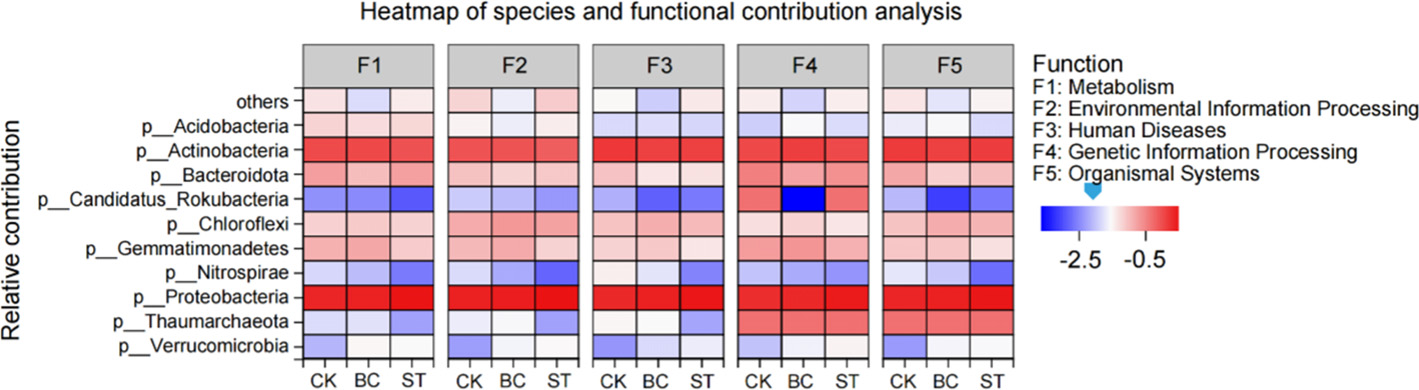
Shows the contributions of soil phosphorus (P) transformation microorganisms to various functions under biochar (BC) and straw (ST) treatments compared to the control (CK) with long-term saline water drip irrigation. CK, control under saline drip irrigation; BC, biochar addition; ST, straw addition.

Further analysis of microbial contributions to soil phosphorus (P) transformation functions (**Figure 6**) revealed that Proteobacteria exhibited the highest contribution across all functions, followed by Actinobacteria, Bacteroidota, and Gemmatimonadetes. The application of both biochar (BC) and straw return (ST) treatments improved the functional contributions of Proteobacteria, Chloroflexi, and Verrucomicrobia compared to the control (CK), but resulted in a reduction in the contributions of Nitrospirae and Candidatus Rokubacteria. Additionally, ST treatment enhanced the contribution of Bacteroidota specifically to metabolic functions relative to CK, while decreasing its involvement in other functional categories.

### 3.6 Gene expression in P transformation

**Figure 7:**
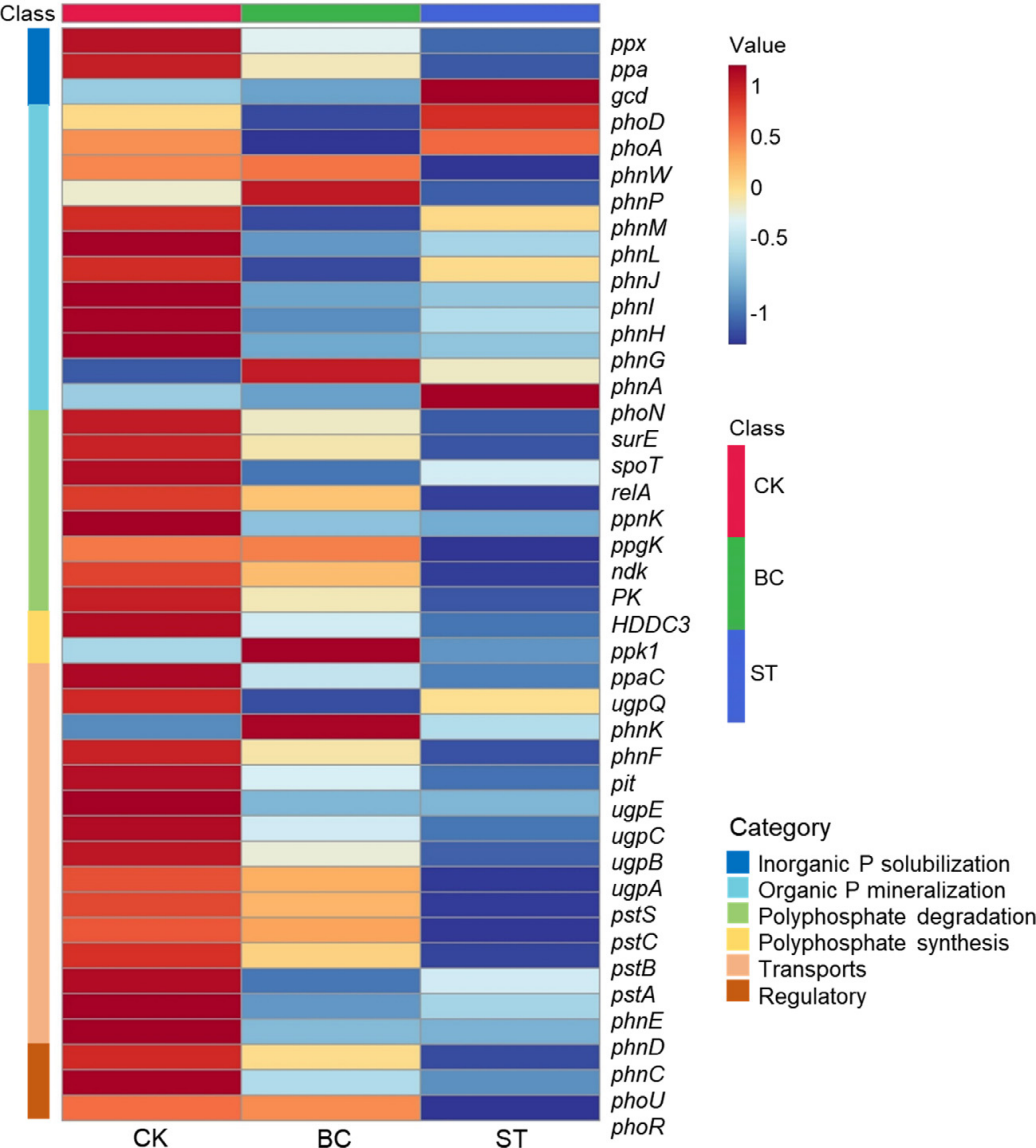
Heat map of functional genes of soil P transformation pathway under biochar and straw returned conditions. CK, long-term saline water drip irrigation; BC, biochar return under long-term saline drip irrigation conditions; ST, straw return under long-term saline drip irrigation conditions.

**Figure 8:**
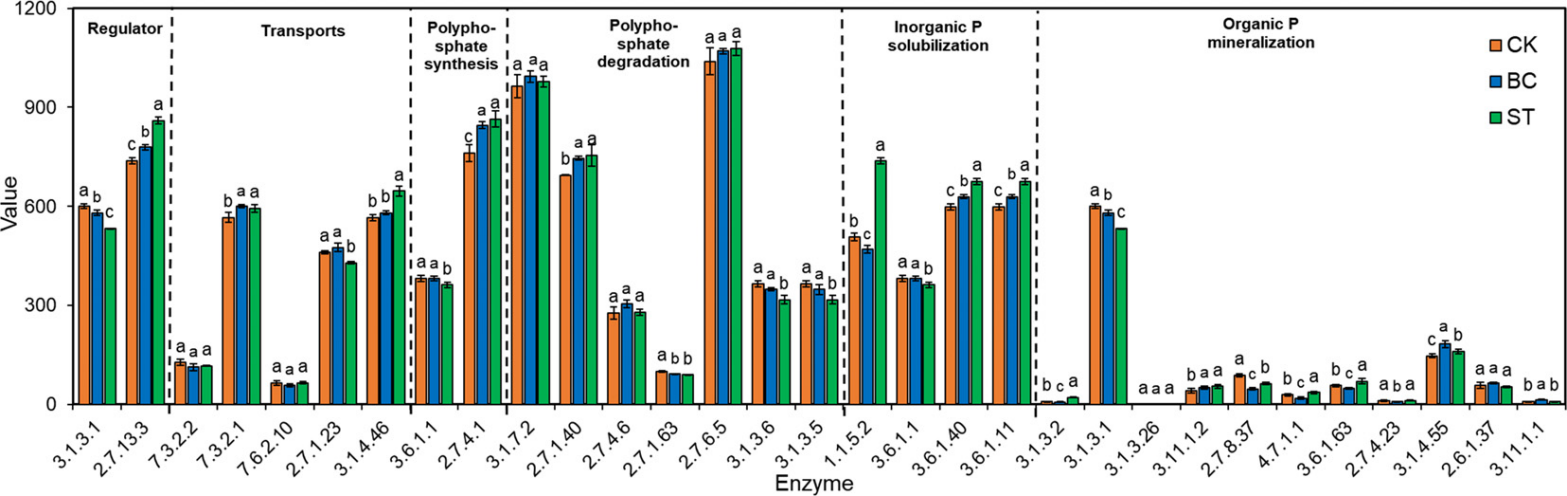
Shows the abundance values of key enzymes involved in soil phosphorus (P) transformation pathways under biochar (BC) and straw return (ST) treatments. Lowercase letters denote significant differences between the means within the same column (*P* < 0.05). Columns with error bars represent mean values ± standard error (n = 3). The horizontal axis displays the enzyme commission (EC) numbers. CK, control with long-term saline drip irrigation; BC, biochar under saline drip irrigation; ST, straw under saline drip irrigation.

Six important enzyme genes involved in the production and degradation of polyphosphates, their transport, regulation, and inorganic P solubilization, organic P mineralization, and inorganic P solubilization were the focus of a thorough macro-genomic investigation carried out to study soil phosphorus (P) transformation processes (**Figures 7**, **8**). Compared to the control (CK), biochar treatment (BC) resulted in a reduction in the expression of the *phoA*, *phoB*, and *phoD* genes, which encode for alkaline phosphatase (EC 3.1.3.1), as well as the *phnG*, *phnH*, *phnI*, and *phnL* genes encoding P-containing group transferase (EC 3.7.8.37), and the *gcd* gene encoding ubiquinone oxidoreductase (EC 1.1.5.2). However, BC enhanced the expression of the *phnP* gene encoding 5-phosphate-α-D-ribose 1,2-cyclic phosphate hydrolase (EC 3.1.4.55) and the *phnX* gene encoding 2-oxoethylphosphonate phosphonohydrolase (EC 3.11.1.1). In contrast, straw return (ST) decreased *phoB* expression but increased the expression of *phoA* and *phoD*, which also encode alkaline phosphatase. ST also downregulated the expression of *phnG*, *phnH*, *phnI*, and *phnL* genes (P-containing group transferase) and *phnL* (P-monoester hydrolase, EC 3.1.3.5, 3.1.3.6), but upregulated the *gcd* gene (ubiquinone oxidoreductase), *phoN* (acid phosphatase, EC 3.1.3.2), and *phnA* (phosphonoacetic acid phosphonolipase, EC 3.11.1.2).

### 3.7 Soil microbial contribution to functional genes for P transformation

**Figure 9:**
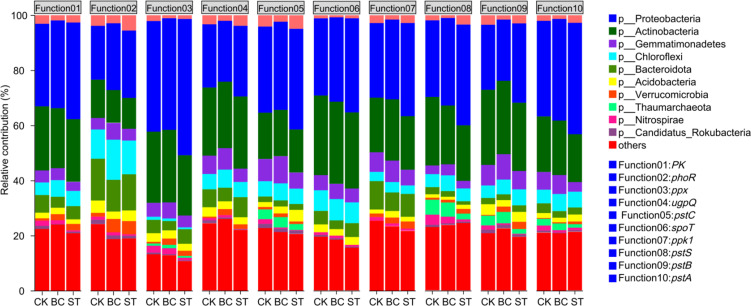
Contribution of soil P transformation microorganisms to functional genes. CK, long-term saline water drip irrigation; BC, biochar return under long-term saline drip irrigation conditions; ST, straw return under long-term saline drip irrigation conditions.

The contributions of soil phosphorus (P) transformation microorganisms to their respective functional genes are illustrated in **Figure 9**. Proteobacteria exhibited the highest contribution to each gene function, followed by Actinobacteria. Proteobacteria’s contribution to the *ugpQ* and *pstB* genes was decreased by applying biochar (BC) as opposed to the control (CK), but it was raised to other functional genes. On the other hand, Proteobacteria’s contribution to all functional genes examined was enhanced by straw return (ST). In comparison to CK, BC for Actinobacteria increased its contribution to the *ppx, pstC, spoT*, and *ppk1* genes while decreasing its contribution to the other genes. Meanwhile, ST elevated Actinobacteria’s contribution to the *ugpQ* gene but reduced its involvement in other functional genes.

### 3.8 Microbial genes and phosphorus correlations

**Figure 10:**
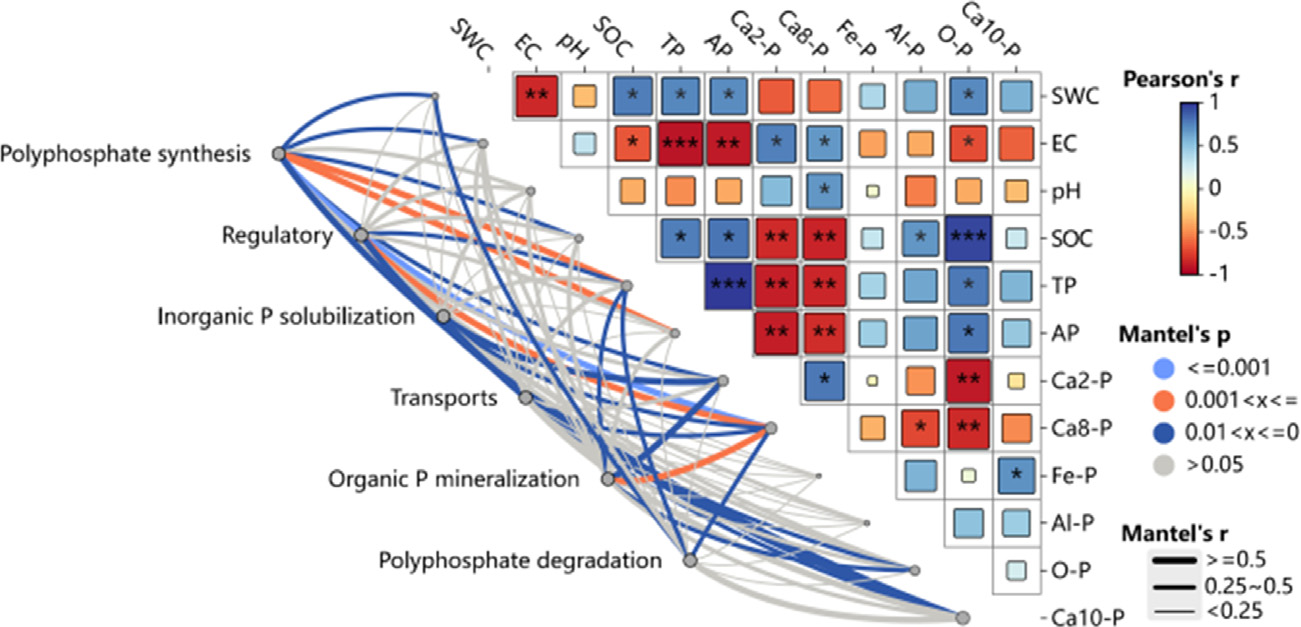
Illustrates the correlation between soil phosphorus (P) transformation microbial communities and soil environmental factors. Red and blue colors indicate positive and negative correlations, respectively. Asterisks (*) denote significant correlations (*P* < 0.05), while double asterisks (**) indicate highly significant correlations (*P* < 0.01). Triple (***) asterisks indicate extremely significant correlations (P < 0.001). CK—control under long-term saline drip irrigation; BC—biochar amendment under saline drip irrigation; ST—straw amendment under saline drip irrigation.

**Figure 11:**
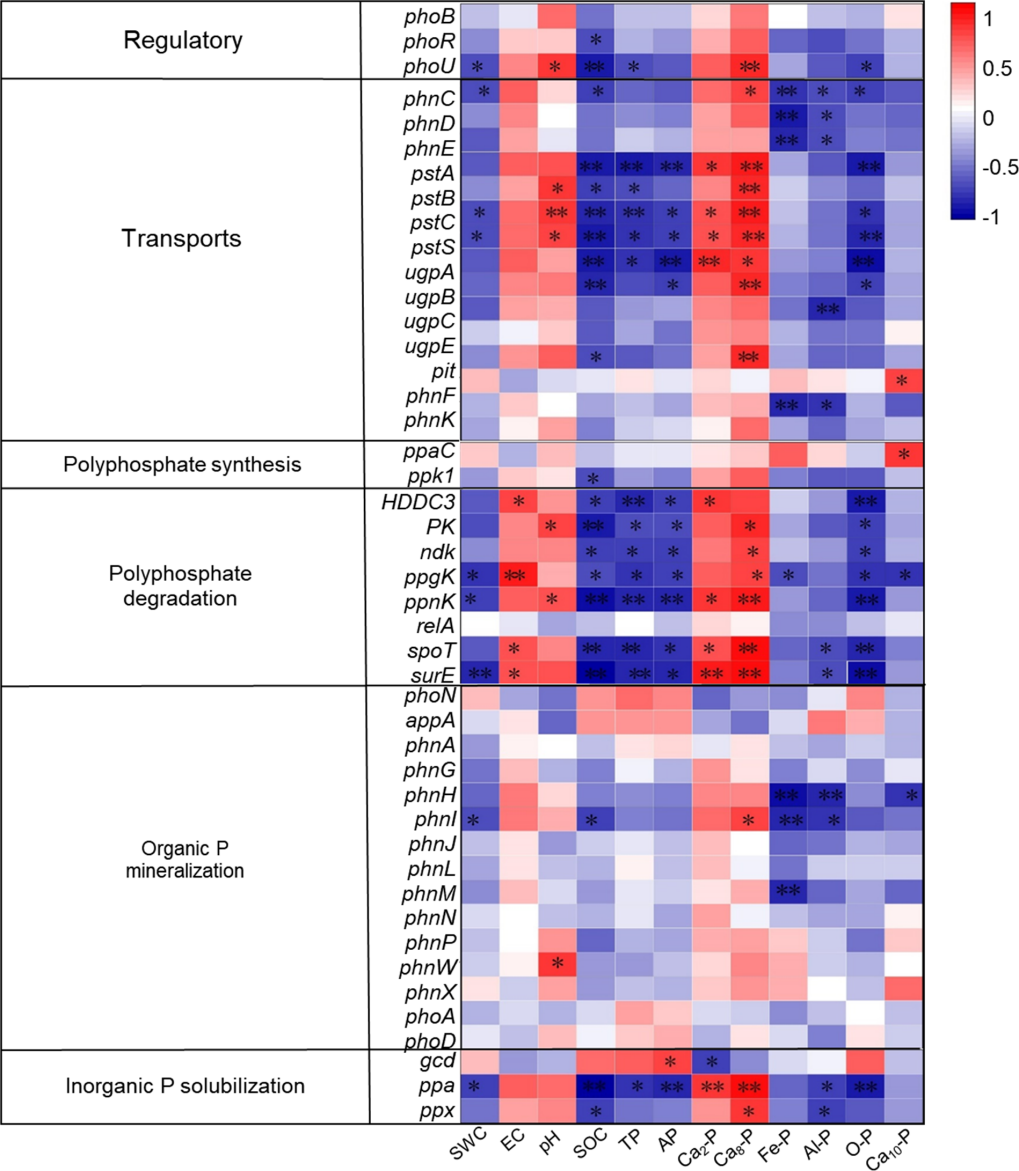
Presents a correlation matrix between soil phosphorus (P) transformation functional genes and soil environmental factors. Red and blue colors represent positive and negative correlations, respectively. Single asterisks (*) indicate significant correlations (*P* < 0.05), while double asterisks (**) denote highly significant correlations (*P* < 0.01). CK—control under long-term saline drip irrigation; BC—biochar amendment under saline drip irrigation; ST—straw amendment under saline drip irrigation.

The physicochemical characteristics of soil, as well as the amounts of inorganic phosphorus (P) fractions, are strongly linked to the existence and activity of microbial genes related to the breakdown and synthesis of polyphosphate, organic P mineralization, inorganic P solubilization, regulation, and transport (**Figure 10**). More specifically (**Figure 11**), the abundance of genes encoding regulatory functions (*phoU*), transport proteins (*phnC*, *pstC*, and *pstS*), polyphosphate degradation (*ppgK*, *ppnK*, and *surE*), organic P mineralization (*phnI*), and inorganic P solubilization (*ppa*) showed a consistent and significant negative correlation with soil water content (SWC). Conversely, soil electrical conductivity (EC) was significantly and positively correlated with genes encoding available P (*HDDC3*, *ppgK*, *spoT*, and *surE*). Soil pH displayed significant positive correlations with genes encoding regulatory (*phoU*), transport (*pstB*, *pstC*, and *pstS*), polyphosphate degradation (*PK* and *ppnK*), and organic P mineralization (*ppnW*).

The abundance of genes encoding transport (*pstA*, *pstC*, *pstS*, and *ugpA*), polyphosphate degradation (*ppnK*, *spoT*, and *surE*), and inorganic P solubilization (*ppa*) was significantly and negatively correlated with soil organic matter (OM), total phosphorus (TP), available phosphorus (AP), and occluded P (O-P), but positively correlated with soil calcium-bound P fractions (*Ca_2_-P* and *Ca_8_-P*). Additionally, soil Fe-P and Al-P were significantly negatively correlated with genes encoding transport proteins (*phnC*, *phnD*, and *phnE*) and organic P mineralization (*phnH* and *phnI*). Genes associated with polyphosphate degradation (*ppgK*) and organic P mineralization (*phnH*) were negatively connected with soil Ca_8_-P, but genes involved in transport (*phnF*) and polyphosphate synthesis (*ppaC*) showed a substantial positive connection.

**Figure 12:**
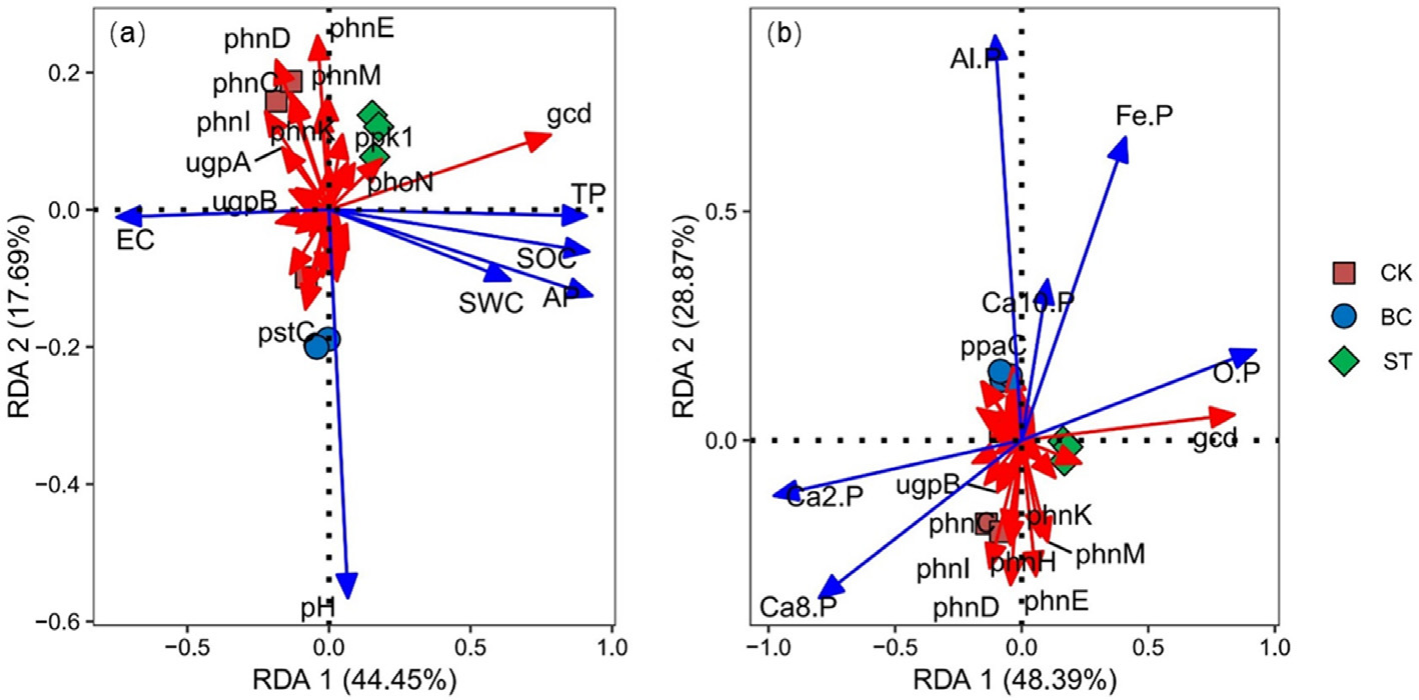
RDA analysis between soil P transformation functional genes and environmental factors. CK, long-term saline water drip irrigation; BC, biochar return under long-term saline drip irrigation conditions; ST, straw return under long-term saline drip irrigation conditions. In the figures, **(A)** represents the correlation analysis between functional genes and physicochemical properties, while **(B)** represents the correlation analysis between functional genes and inorganic phosphorus components.

The Redundancy Analysis (RDA) of functional genes and environmental factors (**Figure 12**) revealed that axes 1 and 2 explained 44.45% and 17.69% of the total variation in functional genes in **Figure 12A**, and 48.39% and 28.87% in **Figure 12B**, respectively. Both treatments separated the biochar (BC) from the control (CK) along axis 2, and the straw return (ST) treatment from the CK along axis 1, suggesting that both had a substantial impact on the functional genes involved in soil P transformation. The RDA also showed that functional gene variation was significantly associated with Ca_2_-P (39.3% explained, *P* = 0.002) and Al-P (13.1% explained, *P* = 0.024) (**Figure 12B**). Specifically, the *ppaC* gene was positively correlated with Al-P, whereas the *ugpB*, *phnG*, *phnI*, *phnD*, *phnH*, *phnE*, *phnK*, and *phnM* genes showed significant negative correlations with Al-P.

### 3.9 Enhancing phosphorus cycling in saline soils with biochar and straw

**Figure 13:**
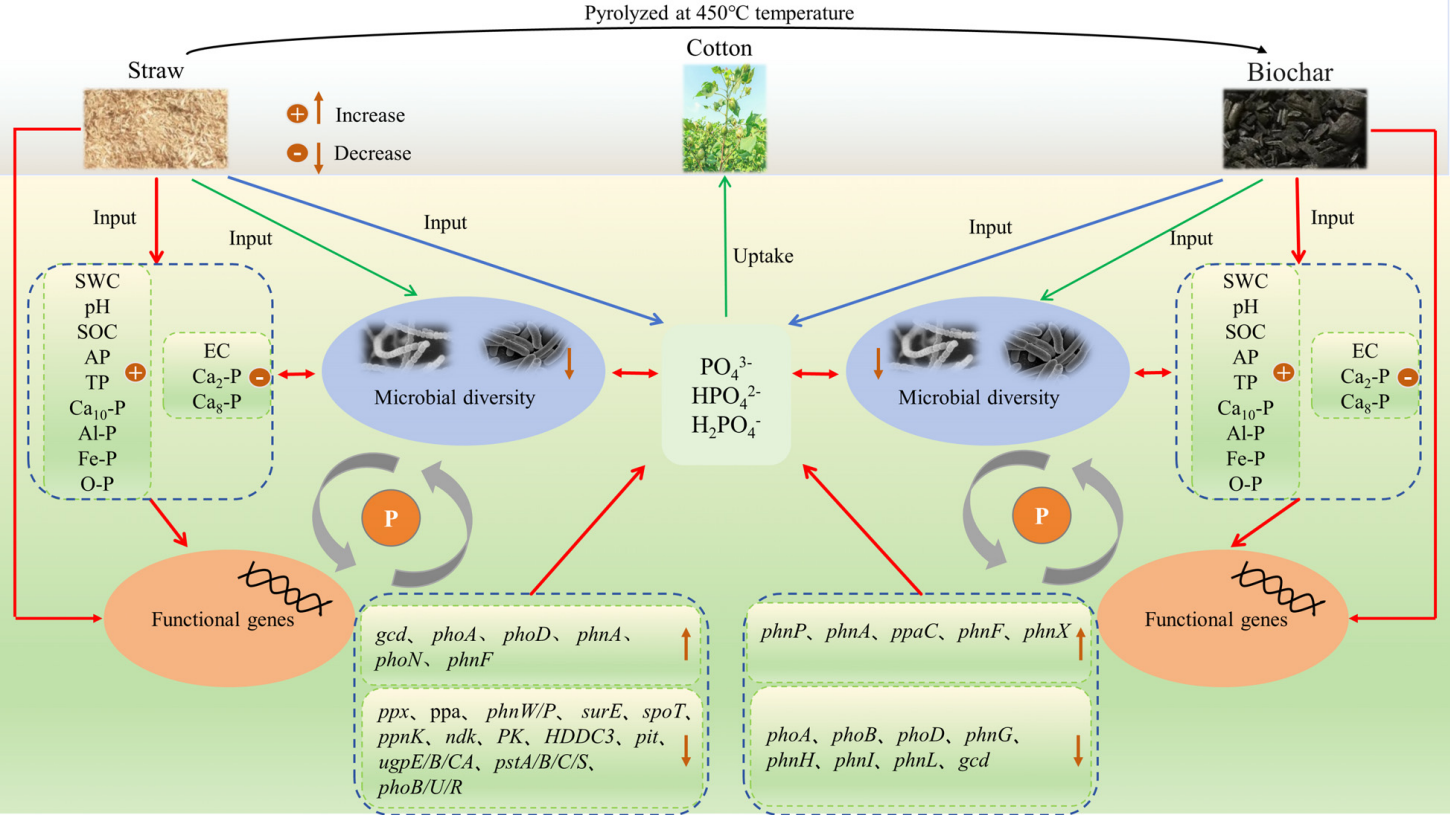
Microecological Mechanisms of biochar and straw influence on soil phosphorus transformation in saline water drip-irrigated cotton fields.

The application of biochar and straw can effectively regulate the cycling process of phosphorus (P) nutrients in soils of cotton fields subjected to long-term saline water drip irrigation. In this study, we compared soil P nutrient cycling under conditions of biochar and straw application, analyzing the differences in soil physicochemical properties and inorganic P components. We also examined the variations in soil P-cycling microbial communities and the trends of functional gene changes from both microbiological and macrogenomic perspectives. This exploration aims to elucidate the regulatory mechanisms of soil P cycling in cotton fields under long-term saline and drip irrigation conditions (**Figure 13**). Specifically, the return of biochar and straw to the soil can mitigate salt accumulation in the surface layer, enhance the physicochemical properties of cotton soils, and increase nutrient availability. The efficiency of soil P nutrients and the soil’s ability to supply P are both enhanced by these additions. Additionally, they stimulate the expression of functional genes linked to phosphorus cycling and affect the activity of microorganisms involved in the soil P cycle, facilitating adaptation to the new environment. Under long-term salty water drip irrigation, after straw and charcoal were once again available, both metrics dropped in the structural diversity of microbial communities involved in soil phosphorus (P) transformation and the number of functional genes. It is crucial to understand that microbial gene sequences are assessed under certain circumstances and at particular times in macrogenomic and microbiome analyses.

## 4 Discussion

### 4.1 Effects of biochar and straw on soil phosphorus dynamics

It has been demonstrated that long-term saline water drip irrigation increases soil electrical conductivity (EC) and reduces soil available phosphorus (AP). However, adding both biochar and straw to the soil under long-term saline water drip irrigation increases soil water content (SWC), soil organic carbon (SOC), total phosphorus (TP), and available phosphorus (AP) levels significantly while lowering soil electrical conductivity (EC). Straw and biochar naturally contain nutrients, and their surfaces’ capacity to absorb charges accounts for the increase in soil nutrients (Aziz et al., 2023). Biochar and straw can serve as phosphorus sources by gradually releasing phosphates through microbial activity, providing P to the soil and enhancing uptake by cotton roots, thereby increasing soil total phosphorus (TP) (Li et al., 2020). The dissolved organic matter in biochar forms organo-metallic complexes with iron and aluminum oxides, affecting metal ion adsorption and phosphorus immobilization, which improves soil available phosphorus (AP) (Xu et al., 2014). The porous structure of biochar adsorbs salt ions, reducing soil electrical conductivity (EC) (Zhang J. et al., 2019), while its binding properties promote agglomeration, enhancing water retention and soil water content (SWC) (Liu R et al., 2024; Blanco-Canqui, 2017; Meyer et al., 2024). Biochar and straw also increase soil organic matter and the carbon pool (Liu et al., 2020; Gu et al., 2022) due to their stable carbon content, which supports carbon sequestration (Aziz et al., 2023), and their high surface area, which adsorbs C and N from the soil. This interaction forms stable organic-inorganic complexes, mitigating carbon release from decomposition (Mukherjee and Lal, 2014; Guo et al., 2023). These findings validate our hypothesis that long-term saline irrigation increases soil salinity and reduces phosphorus (P) availability, while biochar and straw amendments effectively enhance soil phosphorus availability and mitigate salt accumulation.

Our study indicated that biochar amendment significantly increased the content of Al-P, Fe-P, and Ca_10_-P in the soil, while notably reducing the content of Ca_2_-P and C_a8_-P. This suggests that biochar may enhance phosphorus transformation, thereby increasing the proportion of more stable phosphorus pools in the soil (Li et al., 2019). Ca_10_-P is a highly stable phosphorus form, and its formation may be attributed to the alkaline nature and high cation exchange capacity (CEC) of biochar. These properties allow biochar to adsorb large amounts of Ca²^+^ in calcareous soils, forming stable calcium-phosphorus compounds with phosphate, consistent with the findings of Sun et al. (2018). Additionally, the abundant functional groups on the surface of biochar, such as carboxyl and phenolic hydroxyl groups, may promote the formation of Al-P and Fe-P through coordination with Al³^+^ and Fe³^+^ (Wang et al., 2012). This transformation reduces the proportion of highly active Ca_2_-P and Ca_8_-P, thereby decreasing the mobility and loss risk of phosphorus while enhancing the stability and long-term availability of the phosphorus pool. Relevant studies have also pointed out that biochar contains a large amount of P-metal complexes, such as FePO_4_, AlPO_4_, and CaPO_4_, which directly increase the soil contents of Ca-P, Al-P, and Fe-P (Sun et al., 2018). This effect can be attributed to two primary mechanisms: (1) biochar may release more stable phosphorus forms during pyrolysis (Tian et al., 2021); and (2) biochar can adsorb phosphorus from the soil solution, thereby increasing the content of stable phosphorus pools (Xu et al., 2019). This transformation demonstrates that biochar amendment primarily increases the proportion of stable phosphorus pools, which is critical for long-term phosphorus retention and supply in soils but may reduce the short-term bioavailability of phosphorus. Furthermore, our study found that straw return significantly increased the soil contents of Al-P, Fe-P, and O-P while reducing the contents of Ca_2_-P and Ca_8_-P. This aligns with the findings of Zhang et al. (2022), who reported that straw incorporation can enhance the stability of inorganic phosphorus pools. Organic acids released during straw decomposition may be the key mechanism driving this transformation. These organic acids can dissolve highly active Ca_2_-P and Ca_8_-P, promoting their conversion to Al-P, Fe-P, and O-P (Wang et al., 2014). In addition, the increase in soil organic matter due to straw decomposition provides an important foundation for the formation of O-P. The increase in O-P suggests that straw return improves short-term phosphorus supply while significantly enhancing the stability and storage potential of the phosphorus pool (Chang et al., 2024). In summary, under saline water drip irrigation, both biochar and straw return have significant regulatory effects on soil phosphorus transformations and mitigate the impact of salinity on soil phosphorus utilization to some extent. These findings support our hypothesis that biochar and straw return under saline water drip irrigation significantly improve the stability and availability of soil phosphorus pools.

### 4.2 Effects of biochar and straw on soil microbial diversity and phosphorus availability

Microbial communities play a crucial role in regulating soil phosphorus (P) transformation (Amini et al., 2016). The return of biochar and straw significantly impacts the activity and persistence of these microorganisms (Shamim and Joann, 2016; Yang and Lu, 2022). According to our findings, in saline water drip-irrigated cotton fields, the use of biochar and straw led to a decrease in the richness and diversity of the soil microbial community, as shown by decreases in the Ace, Chao, and Shannon indices and an increase in the Simpson index (Shang et al., 2023). The observed increases in soil pH and nutrient levels after the restoration of biochar and straw may be the cause of this loss in microbial diversity. These increases can promote the growth of particular microbial communities (Zhang et al., 2024). Through competition, the synthesis of antibiotics, and other mechanisms, some particular microbes may prevent the growth of others, resulting in a general decline in microbial diversity and abundance. In our study, the dominant phyla among soil P-transforming microorganisms included Proteobacteria, Actinobacteria, and Gemmatimonadetes. Numerous taxonomic analyses have identified Proteobacteria, Actinobacteria, and Gemmatimonadetes as highly adaptable and widely distributed in the soil (Liu et al., 2023). By secreting different enzymes, including phosphatases, that break down complicated organic P molecules into forms that plants can use, these phyla play a critical role in the mineralization of organic P (Li et al., 2022). The relative abundance of Proteobacteria increased in our investigation with both the addition of biochar and straw, supporting findings from (Shang et al., 2023). Some members of the Proteobacteria phylum can reduce soil pH through the secretion of organic acids, which promotes the dissolution of inorganic phosphate and enhances P bioavailability (Shang et al., 2023). Additionally, Actinobacteria produce antibiotics that suppress pathogenic bacteria, thereby indirectly promoting plant health and improving P uptake (Zhang et al., 2024). In this study, the reapplication of biochar and straw decreased the relative abundance of Actinobacteria, which typically thrive under high salt conditions and may proliferate under salt stress (Menasria et al., 2022). Biochar and straw mitigated this effect to some extent. Gemmatimonadetes may either compete or cooperate with other P-solubilizing microbes like Actinobacteria and Ascomycetes in soil P cycling through distinct metabolic pathways (Wang H et al., 2024), highlighting their critical role in phosphorus transformations (Pang et al., 2024). Biochar application significantly reduced Bacteroidota, Acidobacteria, and Planctomycetota while increasing Chloroflexi, Gemmatimonadetes, Thaumarchaeota, and Verrucomicrobia. In contrast, straw return lowered the abundance of Actinobacteria, Gemmatimonadetes, and Thaumarchaeota, but the abundance of Proteobacteria, Planctomycetota and Verrucomicrobia increased significantly. Biochar significantly enhances the abundance of phosphorus-transforming microorganisms, such as Chloroflexi, Gemmatimonadetes, Thaumarchaeota, and Verrucomicrobia, by regulating soil pH, increasing phosphorus availability, and improving soil structure (Zhao et al., 2024). The alkaline nature of biochar helps provide an optimal growth environment, particularly for microorganisms that are more active in neutral to mildly alkaline soils. Additionally, biochar improves soil aeration and water retention, fostering microbial growth and metabolic activity (Wang X et al., 2024). Biochar also promotes the degradation of organic matter, releasing stable carbon sources that supply energy to phosphorus-transforming microorganisms, thereby enhancing their synergistic roles in phosphorus cycling (Jones et al., 2011). For example, Chloroflexi and Gemmatimonadetes may participate in the mineralization of organic phosphorus, while Thaumarchaeota may indirectly influence phosphorus desorption and dissolution through interactions with nitrogen cycling (Wang et al., 2019). Biochar improves the soil’s physical environment, providing a more stable microhabitat that promotes the activity and abundance of these microorganisms. Furthermore, certain species of Chloroflexi and Verrucomicrobia have been shown to secrete phosphatases, and biochar may enhance the activity of these enzymes by providing additional carbon sources, thereby effectively increasing the concentration of available phosphorus in the soil (Hou et al., 2024). The organic carbon sources supplied by straw return promote the proliferation of these microorganisms, particularly through organic phosphorus mineralization and inorganic phosphorus transformation, which increases the availability of phosphorus in the soil (Bai et al., 2024). Proteobacteria degrade organic matter to release phosphate and, through phosphatase secretion or interactions with other microorganisms, enhance phosphorus desorption; Planctomycetota receives abundant organic carbon sources after straw return, enhancing its effective utilization of phosphorus; Verrucomicrobia also releases more available phosphorus through the degradation of organic matter, driving phosphorus mineralization (Liu F et al., 2024). In conclusion, straw return improves the supply of soil organic matter and nutrients, providing an optimal growth environment for phosphorus-transforming microorganisms, thereby enhancing their activity and the biological availability of phosphorus in the soil.

These findings indicate that both biochar and straw significantly altered the microbial communities involved in soil phosphorus (P) transformation, albeit with differences. Biochar promoted the activity of phosphorus-related microorganisms by improving the soil physical environment and providing slowly released nutrients, whereas straw return enhanced microbial metabolic activity through the addition of organic matter, particularly in organic phosphorus mineralization and phosphorus transformation processes. Correlation analysis confirmed a strong relationship between soil P fractions, physicochemical properties, and microbial community composition. Both biochar and straw amendments reshaped the soil microbial community by altering the soil’s physicochemical properties and increasing organic matter, thereby influencing phosphorus cycling and availability. This shift in the soil microbial community may be a key mechanism underlying their promotion of phosphorus transformation processes.

### 4.3 Effects of biochar and straw amendments on soil P dynamics and microbial functionality

Soil phosphorus (P) cycling microbial functional genes provide crucial insights into changes in microbial communities and are essential for understanding soil P dynamics (Widdig et al., 2020). The processes involved in organic P mineralization, inorganic P solubilization, transport, control, and polyphosphate breakdown and synthesis in cotton field soils were examined in this work using macrogenomic analysis. By influencing microbial development and the physicochemical characteristics of the soil, the introduction of biochar and straw changed important P transformation pathways (Zhang P et al., 2022). The inorganic P solubilization pathway was influenced differently by biochar and straw return. Biochar application decreased the content of soil methanol dehydrogenase (EC 1.1.5.2) and its related gene *gcd*, while straw increased both, likely due to the differing carbon availability (Sigmund et al., 2023; Zhang P et al., 2022; Che et al., 2023). Additionally, soil inorganic pyrophosphatase (EC 3.6.1) and dephosphatase (EC 3.6.1.40) levels were elevated by biochar and straw, although the corresponding *ppx* gene was less common. This implies a change in the pathways responsible for the metabolism of P, suggesting that microbes are maximizing their adaptation to the soil environment (Mizukami et al., 2024; Zhi et al., 2023). In the organic P mineralization route, biochar and straw both boosted the activity of soil phosphodiesterase (EC 3.11.1.2). Because of the availability of carbon and energy supplies, biochar increased the *PhnP* gene’s abundance whereas straw lowered it (Turner et al., 2002; Wang et al., 2023). Moreover, because of increased inorganic P and feedback inhibition, biochar and straw decreased alkaline phosphatase (EC 3.1.3.1) (Zhou et al., 2021). Biochar decreased the abundance of *phoA*, *phoB*, and *phoD* genes by raising soil pH and inducing feedback inhibition, while straw enhanced *phoA* and *phoD* expression by supplying organic matter (Cao et al., 2022; Zhou et al., 2021).

These results indicate that biochar and straw significantly reduce the abundance of functional genes associated with soil P transformation. Although biochar improves soil health, it also has complicated regulatory effects on microbial populations involved in soil P cycling. This means that maximizing the use of biochar and straw is essential for enhancing soil management and sustainability. The findings support our theory that biochar and straw modify the microbial populations involved in soil P cycling and induce changes in gene expression to adapt to novel settings. To learn more about these dynamics, long-term monitoring of the P availability in the soil, enzyme activity, and gene expression is required.

This study enhances our understanding of functional genes in P-transforming microorganisms during the amelioration of saline soils with biochar and straw, contributing to our predictive understanding of P-cycling mechanisms in future arid zone soils. To better understand how biochar and straw alter specific microbial functional genes and how they work intrinsically to improve saline soils, future studies should concentrate on these genes. This paper provides a theoretical framework for the development of microbial agents to improve cotton yields, mitigate the problems associated with long-term salt irrigation in arid regions, and modify saline soil conditions.

## 5 Conclusions

This study was performed to investigate the regulatory effects of biochar and straw return on soil phosphorus cycling and microbial functional genes under long-term saline water drip irrigation conditions. We found that the application of biochar and straw significantly reduced the salt accumulation in the surface soil caused by long-term saline irrigation, enhanced the stability and availability of phosphorus in the soil, and decreased the richness and diversity of microbial communities involved in phosphorus cycling, as well as the abundance of functional genes. Biochar return regulated soil phosphorus cycling under long-term saline water drip irrigation by increasing the abundance of Proteobacteria and the gene encoding 5-phospho-*α*-D-ribose 1,2-cyclic phosphate hydrolase (*PhnP*), while decreasing the relative abundance of Actinobacteria, and genes encoding ubiquinone oxidoreductase (*gcd*) and alkaline phosphatase (*phoA*, *phoB*, and *phoD*). Straw return regulated phosphorus cycling under long-term saline water drip irrigation by increasing the abundance of Proteobacteria and alkaline phosphatase genes (*phoA* and *phoD*), while decreasing the relative abundance of Actinobacteria, and genes encoding ubiquinone oxidoreductase (*gcd*) and 5-phospho-*α*-D-ribose 1,2-cyclic phosphate hydrolase (*PhnP*). Among these factors, Ca_2_-P and Al-P were identified as the main contributors to soil phosphorus transformation and microbial community dynamics.

## Data Availability

The original contributions presented in the study are publicly available. This data can be found here: NCBI BioProject, accession PRJNA1200914.

## References

[B1] Aguiar D. Melo V. F. Nogueira M. A. Corrêa R. S. (2024). The role of microbial mechanisms on the availability of soil phosphorus from fixed and structural mineral fractions. J. Soil Sci. Plant Nutr. 1-16, 8192–8207. doi: 10.1007/s42729-024-02106-z

[B2] Ahmad I. Ahmad M. Hussain A. Jamil M. (2021). Integrated use of phosphate-solubilizing Bacillus subtilis strain IA6 and zinc-solubilizing Bacillus sp. strain IA16: a promising approach for improving cotton growth. Folia Microbiologica 66, 115–125. doi: 10.1007/s12223-020-00831-3 33099750

[B3] Amini S. Ghadiri H. Chen C. Marschner P. (2016). Salt-affected soils, reclamation, carbon dynamics, and biochar: a review. J. Soils Sediments 2016, 16: 939–953. doi: 10.1007/s11368-015-1293-1

[B4] Andriamananjara A. Rabeharisoa L. Prud’homme L. Morel C. (2016). Drivers of plant-availability of P from thermally conditioned sewage sludge as assessed by isotopic labeling. Front. Nutr. 3. doi: 10.3389/fnut.2016.00019 PMC4909739 27379240

[B5] Atta K. Mondal S. Gorai S. Singh A. P. Kumari A. Ghosh T.. (2023). Impacts of salinity stress on crop plants: Improving salt tolerance through genetic and molecular dissection. Front. Plant Sci. 14. doi: 10.3389/fpls.2023.1241736 PMC10540871 37780527

[B6] Awaad H. A. Mansour E. Akrami M. Fath H. E. S. Javadi A. A. Negm A. (2020). Availability and feasibility of water desalination as a non-conventional resource for agricultural irrigation in the MENA region: A review. Sustainability. 12, 7592. doi: 10.3390/su12187592

[B7] Aziz S. Shabana B. Mohammad M. H. Partha B. Muhammad I. A. Muhammad B.. (2023). A review on influence of biochar amendment on soil processes and environmental remediation. Biotechnol. Genet. Eng. Rev. 40, 3270–3304. doi: 10.1080/02648725.2022.2122288 36747352

[B8] Bai S. Ge Y. Yao D. Wang Y. Tan J. Zhang S.. (2024). Effect of straw retention and mineral fertilization on P speciation and P-transformation microorganisms in water extractable colloids of a Vertisol. Biogeosciences 2024, 1–44. doi: 10.5194/egusphere-2024-983

[B9] Blanco-Canqui H. (2017). Biochar and soil physical properties. Soil Sci. Soc. America J. 81, 687–711. doi: 10.2136/sssaj2017.01.0017

[B10] Cao N. Zhi M. Zhao W. Pang J. Hu W. Zhou Z.. (2022). Straw retention combined with phosphorus fertilizer promotes soil phosphorus availability by enhancing soil P-related enzymes and the abundance of *phoC* and *phoD* genes. Soil Tillage Res. 220, 105390. doi: 10.1016/j.still.2022.105390

[B11] Chang S. Chen C. Fu Q. L. Zhou A. Hua Z. Zhu F.. (2024). PBAT biodegradable microplastics enhanced organic matter decomposition capacity and CO_2_ emission in soils with and without straw residue. J. Hazardous Materials 480, 135872. doi: 10.1016/i.jhazmat.2024.135872 39305590

[B12] Che W. Piao J. Gao Q. Li X. Li X. Jin F. (2023). Response of soil physicochemical properties, soil nutrients, enzyme activity and rice yield to rice straw returning in highly saline-alkali paddy soils. J. Soil Sci. Plant Nutr. 23, 4396–4411. doi: 10.1007/s42729-023-01358-5

[B13] Chen J. Ma X. M. Lu X. K. Xu H. Chen D. X. Li P.. (2023). Long-term P addition alleviates CO_2_ and N_2_O emissions via altering soil microbial functions in secondary rather primary tropical forests. Environ. pollut. 323, 121295. doi: 10.1016/j.envpol.2023.121295 36822311

[B14] Cheng M. H. Wang H. D. Fan J. L. Wang X. K. Sun X. Yang L.. (2021). Crop yield and water productivity under salty water irrigation: A global meta-analysis. Agric. Water Manage. 256, 107105. doi: 10.1016/j.agwat.2021.107105

[B15] Fraser T. Lynch D. H. Entz M. H. Dunfield K. E. (2015). Linking alkaline phosphatase activity with bacterial *phoD* gene abundance in soil from a long-term management trial. Geoderma 257, 115–122. doi: 10.1016/j.geoderma.2014.10.016

[B16] Gu Y. Y. Zhang H. Y. Liang X. Y. Fu R. Li M. Chen C. J. (2022). Effect of different biochar particle sizes together with bio-organic fertilizer on rhizosphere soil microecological environment on saline-alkali land. Front. Environ. Sci. 10. doi: 10.3389/fenvs.2022.949190

[B17] Guo H. J. Li S. N. Min W. Ye J. Hou Z. A. (2019). Ionomic and transcriptomic analyses of two cotton cultivars (Gossypium hirsutum L.) provide insights into the ion balance mechanism of cotton under salt stress. PloS One 14, e0226776. doi: 10.1371/journal.pone.0226776 31869397 PMC6927655

[B18] Guo X. W. Du S. Y. Guo H. J. Min W. (2023). Long-term saline water drip irrigation alters soil physicochemical properties, bacterial community structure, and nitrogen transformations in cotton. Appl. Soil Ecol. 182, 104719. doi: 10.1016/j.apsoil.2022.104719

[B19] He L. L. Zhao J. Yang S. M. Zhou H. Wang S. Q. Zhao X.. (2020). Successive biochar amendment improves soil productivity and aggregate microstructure of a red soil in a five-year wheat-millet rotation pot trial. Geoderma 376, 114570. doi: 10.1016/j.geoderma.2020.114570

[B20] Hou J. Yi G. Hao Y. Li L. Shen L. Zhang Q.. (2024). The effect of combined application of biochar and phosphate fertilizers on phosphorus transformation in saline-alkali soil and its microbiological mechanism. Sci. Total Environ. 951, 175610. doi: 10.1016/j.scitotenv.2024.175610 39163936

[B21] Hu M. J. Le Y. X. Sardans J. Yan R. B. Zhong Y. Sun D. Y.. (2023). Moderate salinity improves the availability of soil P by regulating P-cycling microbial communities in coastal wetlands. Global Change Biol. 29, 276–288. doi: 10.1111/gcb.16465 36181699

[B22] Hu Y. J. Xia Y. H. Sun Q. Liu K. P. Wang S. Q. Chen X. B.. (2018). Effects of long-term fertilization on *phoD*-harboring bacterial community in Karst soils. Sci. Total Environ. 628, 53–63. doi: 10.1016/j.scitotenv.2018.01.314 29428860

[B23] Ji D. C. Ge L. W. Zwieten L. V. An T. T. Li S. Y. Kuzyakov Y.. (2024). Contrasting effects of maize straw and its biochar on aggregation and soil organic matter stabilization. Plant Soil 495, 221–233. doi: 10.1007/s11104-023-06313-y

[B24] Jiang B. F. Gu Y. C. (1989). A suggested fractionation scheme of inorganic P in calcareous soils. Fertilizer Res. 20, 159–165. doi: 10.1007/BF01054551

[B25] Jones D. L. Murphy D. V. Khalid M. Ahmad W. Edwards-Jones G. DeLuca T. H. (2011). Short-term biochar-induced increase in soil CO_2_ release is both biotically and abiotically mediated. Soil Biol. Biochem. 43, 1723–1731. doi: 10.1016/j.soilbio.2011.04.018

[B26] Lai P. Nabi F. Chen H. Zhao C. Yang G. Chen T.. (2023). The long-term straw returning to paddy land altered the soil phosphate fractions and composition of microbial communities. Eurasian Soil Sci. 56, 502–516. doi: 10.1134/S1064229322602207

[B27] Li M. Hao Y. Yan Z. Kang E. Wang J. Zhang K.. (2022). Long-term degradation from marshes into meadows shifts microbial functional diversity of soil phosphorus cycling in an alpine wetland of the Tibetan Plateau. Land Degradation Dev. 33, 628–637. doi: 10.1002/ldr.4180

[B28] Li H. Li Y. Xu Y. Lu X. (2020). Biochar P fertilizer effects on soil P availability. Chemosphere 244, 125471. doi: 10.1016/j.chemosphere.2019.125471 31809932

[B29] Li F. Y. Liang X. Q. Christophe N. Sun T. Liu F. Arai Y. J. (2019). Effects of biochar amendmentson soi phosphorus transformation in agricultural soils. Adv. Agron. 158, 131–172. doi: 10.1016/bs.agron.2019.07.002

[B30] Liu J. Li F. Y. Liu J. Wang S. Liu H. Ding Y.. (2023). Grazing promotes soil P cycling by enhancing soil microbial functional genes for P transformation in plant rhizosphere in a semi-arid natural grassland. Geoderma 430, 116303. doi: 10.1016/j.geoderma.2022.116303

[B31] Liu F. Qian J. Zhu Y. Wang P. Hu J. Lu B.. (2024). Phosphate solubilizing microorganisms increase soil phosphorus availability: a review. Geomicrobiology J. 41, 1–16. doi: 10.1080/01490451.2023.2272620

[B32] Liu R. Tang M. Luo Z. H. Zhang C. Liao C. Y. Feng S. (2024). Straw returning proves advantageous for regulating water and salt levels, facilitating nutrient accumulation, and promoting crop growth in coastal saline soils. Agronomy 14, 1196. doi: 10.3390/agronomy14061196

[B33] Liu M. L. Wang C. Liu X. L. Lu Y. C. Wang Y. F. (2020). Saline-alkali soil applied with vermicompost and humic acid fertilizer improved macroaggregate microstructure to enhance sat leaching and inhibit nitrogen losses. Appl. Soil Ecol. 156, 103705. doi: 10.1016/j.apsoil.2020.103705

[B34] Lychuk T. E. Moulin A. P. Lemke R. L. Izaurralde R. C. Johnson E. N. Olfert O. O.. (2019). Climate change, agricultural inputs, cropping diversity, and environment affect soil carbon and respiration: a case study in Saskatchewan. Canada. Geoderma 337, 664–678. doi: 10.1016/j.geoderma.2018.10.010

[B35] Menasria T. Monteoliva-Sánchez M. Benhadj M. Benammar L. Boukoucha M. Aguilera M. (2022). Unraveling the enzymatic and antibacterial potential of rare halophilic actinomycetes from Algerian hypersaline wetland ecosystems. J. Basic Microbiol. 62, 1202–1215. doi: 10.1002/jobm.202200085 35945171

[B36] Meyer C. Jeanbille M. Breuil M. C. Bru D. Höfer K. Screpanti C.. (2024). Soil microbial community fragmentation reveals indirect effects of fungicide exposure mediated by biotic interactions between microorganisms. J. Hazardous Materials 470, 134231. doi: 10.1016/j.jhazmat.2024.134231 38598881

[B37] Mizukami C. Mukai M. Wagai R. Kitayama K. (2024). Intraspecific variations in activities of four classes of fine root phosphatases in Quercus serrata, a dominant deciduous oak, occurring across a wide soil phosphorus gradient in Japan. Plant Soil, 1–17. doi: 10.1007/s11104-024-06797-2

[B38] Mukherjee A. Lal R. (2014). The biochar dilemma. Soil Res. 52, 217–230. doi: 10.1071/SR13359

[B39] Pang F. Li Q. Solanki M. K. Wang Z. Xing Y. X. Dong D. F. (2024). Soil phosphorus transformation and plant uptake driven by phosphate-solubilizing microorganisms. Front. Microbiol. 15. doi: 10.3389/fmicb.2024.1383813 PMC11005474 38601943

[B40] Pastore G. Kaiser K. Kernchen S. Spohn M. (2020). Microbial release of apatite-and goethite-bound phosphate in acidic forest soils. Geoderma 370, 114360. doi: 10.1016/j.geoderma.2020.114360

[B41] Pu J. H. Jiang N. Zhang Y. L. Guo L. L. Huang W. J. Chen L. J. (2023). Effects of various straw incorporation strategies on soil P fractions and transformations. Global Change Biol. Bioenergy 15, 88–98. doi: 10.1111/gcbb.13010

[B42] Rath K. M. Rousk J. (2015). Salt effects on the soil microbial decomposer community and their role in organic carbon cycling: A review. Soil Biol. Biochem. 81, 108–123. doi: 10.1016/j.soilbio.2014.11.001

[B43] Shamim G. Joann K. W. (2016). Biochemical cycling of nitrogen and P in biochar-amended soils. Soil Biol. Biochem. 103, 1–15. doi: 10.1016/j.soilbio.2016.08.001

[B44] Shang W. Razavi B. S. Yao S. Hao C. Kuzyakov Y. Blagodatskaya E.. (2023). Contrasting mechanisms of nutrient mobilization in rhizosphere hotspots driven by straw and biochar amendment. Soil Biol. Biochem. 187, 109212. doi: 10.1016/j.soilbio.2023.109212

[B45] Sigmund G. Schmid A. Schmidt H. P. Hagemann N. Bucheli T. D. Hofmann T. (2023). Small biochar particles hardly disintegrate under cryo-stress. Geoderma 430, 116326. doi: 10.1016/j.geoderma.2023.116326

[B46] Sun D. Hale L. Kar G. Soolanayakanahally R. Adl S. (2018). P recovery and reuse by pyrolysis: Applications for agriculture and environment. Chemosphere 194, 682–691. doi: 10.1016/j.chemosphere.2017.12.035 29245134

[B47] Tan H. Barret M. Mooij M. J. Morrissey J. P. Dobson A. Griffiths B.. (2013). Long-term phosphorus fertilisation increased the diversity of the total bacterial community and the *phoD* phosphorus mineraliser group in pasture soils. Biol. Fertility Soils 49, 661–672. doi: 10.1007/s00374-012-0755-5

[B48] Tian J. Kuang X. Tang M. Chen X. Huang F. Cai Y.. (2021). Biochar application under low P input promotes soil organic P mineralization by shifting bacterial *phoD* gene community composition. Sci. Total Environ. 779, 146556. doi: 10.1016/j.scitotenv.2021.146556 34030240

[B49] Turner B. L. McKelvie I. D. Haygarth P. M. (2002). Characterisation of water-extractable soil organic phosphorus by phosphatase hydrolysis. Soil Biol. Biochem. 34, 27–35. doi: 10.1016/s0038-0717(01)00144-4

[B50] Vermue E. Metselaar K. van der Zee S.E.A.T.M. (2013). Modeling of soil salinity and halophyte crop production. Environ. Exp. Bot. 92, 186–196. doi: 10.1016/j.envexpbot.2012.10.004

[B51] Wan B. Huang R. Diaz J. M. Tang Y. (2022). Rethinking the biotic and abiotic remineralization of complex phosphate molecules in soils and sediments. Sci. Total Environ. 833, 155187. doi: 10.1016/j.scitotenv.2022.155187 35421464

[B52] Wang T. Camps-Arbestain M. Hedley M. (2014). The fate of P of ash-rich biochars in a soil-plant system. Plant Soil 375, 61–74. doi: 10.1007/s11104-013-1938-z

[B53] Wang T. Camps-Arbestain M. Hedley M. Bishop P. (2012). Predicting phosphorus bioavailability from high-ash biochars. Plant Soil 357, 173–187. doi: 10.1007/s11104-012-1131-9

[B54] Wang H. Chen J. Ruan Y. Sun W. Wang S. Wang H.. (2024). Metagenomes reveal the effect of crop rotation systems on phosphorus cycling functional genes and soil phosphorus avail-ability. Agriculture Ecosyst. Environ. 364, 108886. doi: 10.1016/j.agee.2024.10888

[B55] Wang B. Qin W. Ren Y. Zhou X. Jung M. Y. Han P.. (2019). Expansion of Thaumarchaeota habitat range is correlated with horizontal transfer of ATPase operons. ISME J. 13, 3067–3079. doi: 10.1038/s41396-019-0493-x 31462715 PMC6863869

[B56] Wang X. Riaz M. Babar S. Eldesouki Z. Liu B. Xia H.. (2024). Alterations in the composition and metabolite profiles of the saline-alkali soil microbial community through biochar application. J. Environ. Manage. 352, 120033. doi: 10.1016/j.jenvman.2024.120033 38218168

[B57] Wang C. Wang D. Li Y. Liu S. (2023). Metagenomics of the effect of long-term straw return on the phosphorus cycle in meadow black soil. Agronomy 13, 3003. doi: 10.3390/agronomy13123003

[B58] Widdig M. Heintz-Buschart A. Schleuss P. M. Guhr A. Borer E. T. Seabloom E. W.. (2020). Effects of nitrogen and phosphorus addition on microbial community composition and element cycling in a grassland soil. Soil Biol. Biochem. 151, 108041. doi: 10.1016/j.soilbio.2020.108041

[B59] Xu M. Gao P. Yang Z. Su L. Wu J. Yang G.. (2019). Biochar impacts on P cycling in rice ecosystem. Chemosphere 225, 311–319. doi: 10.1016/j.chemosphere.2019.03.069 30884292

[B60] Xu G. Sun J. N. Shao H. B. Chang S. X. (2014). Biochar had effects on P sorption and desorption in three soils with differing acidity. Ecol. Eng. 62, 54–60. doi: 10.1016/j.ecoleng.2013.10.027

[B61] Yang C. Lu S. (2022). Straw and straw biochar differently affect P availability, enzyme activity and microbial functional genes in an Ultisol. Sci. total Environ. 805, 150325. doi: 10.1016/j.scitotenv.2021.150325 34537703

[B62] Zeng Q. Wu X. Wen X. (2016). Effects of soluble phosphate on phosphate-solubilizing characteristics and expression of *gcd* gene in Pseudomonas frederiksbergensis JW-SD2. Curr. Microbiol. 72, 198–206. doi: 10.1007/s00284-015-0938-z 26573634

[B63] Zhang J. Bai Z. Huang J. Hussain S. Zhao F. Zhu C.. (2019). Biochar alleviated the salt stress of induced saline paddy soil and improved the biochemical characteristics of rice seedlings differing in salt tolerance. Soil Tillage Res. 195, 104372. doi: 10.1016/j.still.2019.104372

[B64] Zhang W. W. Wang C. Xue R. Wang L. J. (2019). Effects of salinity on the soil microbial community and soil fertility. J. Integr. Agriculture. 18, 1360–1368. doi: 10.1016/S2095-3119(18)62077-5

[B65] Zhang N. Y. Wang Q. Zhan X. Y. Wu Q. H. Huang S. M. Zhu P.. (2022). Characteristics of inorganic P fractions and their correlations with soil properties in the non-acidic soils. J. Integr. Agric. 21, 3626–3636. doi: 10.1016/j.jia.2022.08.012

[B66] Zhang P. Xue B. Jiao L. Meng X. Zhang L. Li B.. (2022). Preparation of ball-milled phosphorus-loaded biochar and its highly effective remediation for Cd-and Pb-contaminated alkaline soil. Sci. Total Environ. 813, 152648. doi: 10.1016/j.scitotenv.2021.152648 34963592

[B67] Zhang J. Ye L. Chang J. Wang E. Wang C. Zhang H.. (2024). Straw soil conditioner modulates key soil microbes and nutrient dynamics across different maize developmental stages. Microorganisms 12, 295. doi: 10.3390/microorganisms12020295 38399698 PMC10893213

[B68] Zhao W. Zhao H. Sun X. Wang H. Sun Y. Liang Y.. (2024). Biochar and wood vinegar altered the composition of inorganic phosphorus bacteria community in saline-alkali soils and promoted the bioavailability of phosphorus. J. Environ. Manage. 370, 122501. doi: 10.1016/j.jenvman.2024.122501 39299129

[B69] Zhi R. Deng J. Xu Y. Xu M. Zhang S. Han X.. (2023). Altered microbial P cycling genes drive P availability in soil after afforestation. J. Environ. Manage. 328, 116998. doi: 10.1016/j.jenvman.2022.116998 36516705

[B70] Zhou Y. Zhang T. Jin S. Chen S. Zhang Y. (2021). Effects of Escherichia coli alkaline phosphatase PhoA on the mineralization of dissolved organic phosphorus. Water 13, 3315. doi: 10.3390/w13233315

[B71] Zhu M. Wang Q. Sun Y. Zhang G. (2021). Effects of oxygenated brackish water on germination and growth characteristics of wheat. Agric. Water Manage. 245, 106520. doi: 10.1016/j.agwat.2020.106520

